# A Genomic DNA Reporter Screen Identifies Squalene Synthase Inhibitors That Act Cooperatively with Statins to Upregulate the Low-Density Lipoprotein Receptor[Fn FN3]

**DOI:** 10.1124/jpet.116.239574

**Published:** 2017-06

**Authors:** Alastair G. Kerr, Lawrence C. S. Tam, Ashley B. Hale, Milena Cioroch, Gillian Douglas, Sarina Agkatsev, Olivia Hibbitt, Joseph Mason, James Holt-Martyn, Carole J. R. Bataille, Graham M. Wynne, Keith M. Channon, Angela J. Russell, Richard Wade-Martins

**Affiliations:** Departments of Physiology, Anatomy, and Genetics (A.G.K., L.C.S.T., M.C., S.A., O.H., J.H.-M., R.W.-M.) and Pharmacology (A.J.R.), University of Oxford, Oxford, United Kingdom; Division of Cardiovascular Medicine, British Heart Foundation Centre of Research Excellence, University of Oxford, John Radcliffe Hospital, Oxford, United Kingdom (A.B.H., G.D., K.M.C.); and Chemistry Research Laboratory, Department of Chemistry, University of Oxford, Oxford, United Kingdom (J.M., C.J.R.B., G.M.W., A.J.R.)

## Abstract

Hypercholesterolemia remains one of the leading risk factors for the development of cardiovascular disease. Many large double-blind studies have demonstrated that lowering low-density lipoprotein (LDL) cholesterol using a statin can reduce the risk of having a cardiovascular event by approximately 30%. However, despite the success of statins, some patient populations are unable to lower their LDL cholesterol to meet the targeted lipid levels, due to compliance or potency issues. This is especially true for patients with heterozygous familial hypercholesterolemia who may require additional upregulation of the low-density lipoprotein receptor (LDLR) to reduce LDL cholesterol levels below those achievable with maximal dosing of statins. Here we identify a series of small molecules from a genomic DNA reporter screen that upregulate the LDLR in mouse and human liver cell lines at nanomolar potencies (EC_50_ = 39 nM). Structure-activity relationship studies carried out on the lead compound, OX03771 [(*E*)-*N*,*N*-dimethyl-3-(4-styrylphenoxy)propan-1-amine], led to the identification of compound OX03050 [(*E*)-3-(4-styrylphenoxy)propan-1-ol], which had similar potency (EC_50_ = 26 nM) but a much-improved pharmacokinetic profile and showed in vivo efficacy. Compounds OX03050 and OX03771 were found to inhibit squalene synthase, the first committed step in cholesterol biosynthesis. These squalene synthase inhibitors were shown to act cooperatively with statins to increase LDLR expression in vitro. Overall, we demonstrated here a novel series of small molecules with the potential to be further developed to treat patients either alone or in combination with statins.

## Introduction

Cardiovascular disease remains one of the largest health and economic burdens in the world. High circulating levels of low-density lipoprotein (LDL) cholesterol remain one of the biggest risk factors for cardiovascular disease, because this leads to the accelerated development of atherosclerosis and progression into coronary heart disease (Lusis, 2000). The front-line pharmacotherapies to lower LDL cholesterol are the 3-hydroxy-3-methylglutaryl coenzyme A reductase (HMGCR) inhibitors, known as statins. Statins have been very successful in primary and secondary prevention of coronary heart disease (Pedersen, 1994; Shepherd et al., 1995), with many large randomized control trials demonstrating a reduction in cardiovascular disease directly correlated with lowering of LDL cholesterol (Sniderman et al., 2012). Despite the success of statins, some patients either fail to achieve their lipid goal or experience intolerable side effects such as myotoxicity (Moßhammer et al., 2014), leading them to discontinue taking statin medication. However, there has been some controversy regarding the rate of adverse events related to statins, with some large randomized controlled trials describing no difference in reported side effects compared with placebo (Ridker et al., 2008). The rate of myopathy in these trials is usually around 3% (Bays, 2006), although the rate of statin intolerance in the general population may be as high as 10%–15% (Grundy, 2002; Joy and Hegele, 2009). These side effects are much more common in patients taking the maximum dose of statins, so therapies that could be used in conjunction with a reduced dose of statins to lower LDL cholesterol may increase compliance and help more patients achieve their targeted lipid levels.

Squalene synthase is involved in the same cholesterol biosynthesis pathway as HMGCR but represents the first committed step in cholesterol synthesis. Statins act upstream of squalene synthase and so also inhibit the production of other nonsteroidal isoprenoid molecules, such as isopentenyl adenine (protein synthesis), coenzyme Q_10_ (mitochondrial respiration), and dolichol (glycosylation), a consequence that has been linked to their side effects (Marcoff and Thompson, 2007; Bitzur et al., 2013). Squalene synthase inhibitors have been shown to upregulate the low-density lipoprotein receptor (LDLR) like statins but without inhibiting the production of these other intermediates (Davidson, 2007). In fact, squalene synthase inhibitors administered with statins were shown to have a protective effect on statin-induced myotoxicity (Nishimoto et al., 2007). Many squalene synthase inhibitors have shown similar or greater antihyperlipidemic effects than statins (Ugawa et al., 2000; Nishimoto et al., 2003) and they continue to be developed as promising lipid-lowering agents to complement statins (Ichikawa et al., 2013). The squalene synthase inhibitor lapaquistat acetate reached phase II/III clinical trials (Stein et al., 2011), where it was generally well tolerated and significantly reduced LDL cholesterol. When used in conjunction with statins, lapaquistat acetate reduced LDL cholesterol by a further 19%, compared with statin monotherapy. Unfortunately, lapaquistat acetate was withdrawn due to hepatotoxicity seen in two patients receiving a high dose. It was never established whether the hepatotoxicity was a result of off-target effects of lapaquistat acetate or through inhibition of squalene synthase. Interestingly, squalene synthase knockout mice exhibit only a modest liver injury despite a complete loss of enzyme function, thought to result from a build up of the squalene synthase substrate farnesyl diphosphate (FPP) (Nagashima et al., 2015). Treatment with a statin would alleviate this build up, similar to that already observed using this combination of inhibitors to prevent nonsterol isoprenoid depletion associated with statins (Wasko et al., 2011).

Here we present a set of novel stilbenoid-derived small molecules that can upregulate the LDLR at the mRNA and protein levels with nanomolar potencies. We carried out preliminary structure-activity relationship (SAR) studies to improve the efficacy and pharmacokinetic profile. The mechanism of action was established through enzymatic assays and supported by in silico modeling. We demonstrate that these small molecules are able to drive expression of the human *LDLR* promoter in vivo and when dosed in combination with statins give a much greater effect than can be seen with either inhibitor alone.

## Materials and Methods

### 

#### Cell Culture.

Human Hep3B cells were a kind gift from Dr. Zoe Holloway (University of Oxford, Oxford, UK). Mouse hepatoma Hepa1-6 cells were a kind gift from Dr. Natalia Sacilotto (University of Valencia, Valencia, Spain). Both cell lines were grown in Dulbecco’s modified Eagle’s medium supplemented with 10% fetal bovine serum, 1% penicillin/streptomycin, and 1% l-glutamine in a 5% CO_2_ incubator at 37°C. For mRNA analysis, Hep3B cells were seeded in 24-well plates. For Western blot analysis Hepa1-6 cells were seeded in six-well plates. After 24 hours, cells were changed to Dulbecco’s modified Eagle’s medium supplemented with 5% lipoprotein-deficient serum, 1% penicillin/streptomycin, and 1% l-glutamine. Chinese hamster ovary (CHO) wild-type cells transfected with *pLDLR-Luc* (CHO-*pLDLR-Luc*) establishing a clonal cell line were grown in Ham’s F-12 medium supplemented with 10% fetal bovine serum, 1% penicillin/streptomycin, and 1% l-glutamine in a 5% CO_2_ incubator at 37°C. CHO-*pLDLR-Luc* cells were seeded in 24-well plates. After 24 hours, cells were changed to Ham’s F-12 supplemented with 5% lipoprotein-deficient serum, 1% penicillin/streptomycin, and 1% l-glutamine. Compounds (Compound synthesis [Supplemental methods and materials]), simvastatin (Sigma-Aldrich, St. Louis, MO) and pravastatin (Sigma-Aldrich) dissolved in dimethylsulfoxide, and cholesterol (Sigma-Aldrich) and 25-hydroxycholesterol (Sigma-Aldrich) dissolved in ethanol were added 24 hours after.

#### Luciferase Assay.

CHO-p*LDLR-Luc* cells were lysed 48 hours after compound treatment using lysis buffer containing 1% Triton X-100. 2 mM ATP, 2 mM dithiothreitol, and 1 mM d-luciferin were added to the lysate in luciferase assay buffer containing 15 mM MgSO_4_, 15 mM KPO_4_, and 4 mM EGTA at pH 7.8. Luciferase activity was quantified on a Dynex Technologies MLX 96-well plate luminometer (Dynex Technologies, Chantilly, VA). The protein concentration was determined using a BCA protein assay kit (Thermo Fisher Scientific, Waltham, MA) with bovine serum albumin to generate a standard curve. Luciferase activity was normalized to total protein within each well.

#### Quantitative Real-Time Polymerase Chain Reaction.

RNA was extracted from Hep3B cells 24 hours after compound treatment, using the RNeasy mini kit (Qiagen, Valencia, CA) according to the manufacturer's protocol. cDNA was reverse transcribed from 1 *µ*g total RNA using random primers, SuperScript III Reverse Transcriptase (Thermo Fisher Scientific), and RNaseOUT recombinant RNase inhibitor (Thermo Fisher Scientific). Quantitative real-time polymerase chain reaction was performed on a StepOnePlus Real-Time PCR system with SYBR Green PCR Master Mix (both Thermo Fisher Scientific) according to the manufacturer’s protocol. The following gene- and species-specific primers were used: for LDLR, gacagatgcgaaagaaacga (forward) and acagacaagcacgtctcctg (reverse); and for *β*-actin, agcgcggctacagcttca (forward) and cgtagcacagcttctccttaatgtc (reverse). *β*-actin was used as a housekeeping gene and all samples were run in triplicate. The ∆∆ threshold cycle was calculated and used for quantification.

#### Western Blot Analysis.

Hepa1-6 cells were lysed 48 hours after compound treatment using lysis buffer containing 0.5% NP40. The protein concentration was determined using a BCA protein assay kit with bovine serum albumin to generate a standard curve. Fifteen micrograms of total protein was heated at 90°C for 5 minutes and then loaded into each well and run on 10% SDS-polyacrylamide. Samples were transferred to a polyvinylidene fluoride membrane cut and stained with LDLR (ab30532; Abcam, Cambridge, MA) or *β*-actin (ab8226; Abcam) primary antibody overnight at 4°C. Membranes were then incubated with horseradish peroxidase–conjugated polyclonal rabbit IgG secondary antibody (Abcam). All blots were developed using an enhanced chemiluminescence substrate kit (Pierce/Thermo Scientific) and were exposed to Fuji X-ray films (Fujifilm, Tokyo, Japan) in a dark-room facility. Protein band intensities were quantified by scanning with a Canon M550 scanner (Canon, Melville, NY) and were analyzed using ImageJ software (National Institutes of Health, Bethesda, MD).

#### Adenylate Kinase Assay.

Cell media were aspirated from wells of CHO-p*LDLR-Luc* cells treated with compounds for 48 hours. The adenylate kinase concentration present in the media was quantified using a bioluminescence cytotoxicity assay kit (MBL, Woburn, MA) per the manufacturer’s instructions. Luciferase activity was quantified on a Dynex Technologies MLX 96-well plate luminometer.

#### Squalene Synthase Activity Assay.

Squalene synthase activity was assessed as described earlier (Amin et al., 1992). Briefly, each assay was in 1 ml assay buffer (50 mM phosphate buffer, pH 7.4, containing 10 mM MgCl_2_) containing 0.5 mM NADPH, 12 *µ*g human liver microsomes, and compound or vehicle (dimethylsulfoxide) alone in 16-mm ×100-mm glass screw-cap tubes. All components were allowed to equilibrate for 10 minutes at 37°C before the addition of [^3^H]-FPP (50 nM 0.045 Ci/mmol; PerkinElmer, Wokingham, UK) for a further 10 minutes at 37°C. The reaction was stopped by the addition of 1 ml 15% KOH dissolved in EtOH. Tubes were incubated at 65°C for 30 minutes and then 5 ml petroleum ether was added and shaken for 10 minutes. The lower aqueous phase was frozen and the upper organic phase was transferred to clean glass tubes containing 2 ml distilled water. Then, 1.5 ml upper organic phase was removed and counted with 3 ml scintillation liquid on a Tri-Carb 2800 TR Liquid Scintillation Analyzer (PerkinElmer).

#### Animals and Treatment Protocols.

Animal maintenance and experiments were carried out in accordance with the UK Home Office regulations under the Animals (Scientific Procedures) Act of 1986. Animals had access to food and water ad libitum. All mice were fed a chow diet (Global 16% Protein Rodent Diet, TD.2016; Harlan-Teklad/Envigo, Huntingdon, UK) and maintained at 22°C and 60%–70% humidity, with a regular 12-hour light/dark cycle. Male MF-1 and CD-1 mice weighing between 21 and 25 g were ordered from Charles River Laboratories (Wilmington, MA).

#### Plasma Concentration Analysis of Compound OX03050 Using High-Performance Liquid Chromatography.

CD-1 male mice were administered compound OX03050 [(*E*)-3-(4-styrylphenoxy)propan-1-ol] (40 mg/kg) or vehicle intraperitoneally. Mice were then euthanized at 5, 10, 30, 180, and 360 minutes after compound delivery and plasma was harvested for high-performance liquid chromatography (HPLC) analysis. A standard curve was made up of known concentrations spiked into plasma and ran alongside unknown drug concentration samples each time. To make the standard curve, known compound OX03050 concentrations were spiked into drug-free plasma. Acetonitrile was added to each sample in a 1:8 ratio, and samples were shaken for 15 minutes before being spun at 13,000 rpm for 15 minutes at 4°C. Two-hundred microliters of the supernatant was run on a Waters 600 controller HPLC machine with a C18 reverse-phase column (Waters, Billerica, MA), with a linear gradient mobile phase starting at 20:1 H_2_O with 0.1% trifluoroacetic acid (TFA)/acetonitrile with 0.1% TFA increasing to 1:20 H_2_O with 0.1% TFA/acetonitrile with 0.1% TFA over 7 minutes at a flow rate of 1.5 ml/min.

#### Hydrodynamic Delivery.

Mice weighing between 18 and 35 g received hydrodynamic tail-vein injections of plasmid DNA as described (Liu, Song and Liu, 1999). Animals were anesthetized with isoflurane and their body temperature was maintained using a heating pad. Fifty micrograms of plasmid DNA was resuspended in TransIT-EE Hydrodynamic Delivery Solution (Mirus, Madison, WI), to a total volume equivalent to 10% of the mouse’s total body weight. Delivery of the plasmid was administered via the tail vein with an injection time between 8 and 14 seconds depending on the weight of the mouse. Animals were allowed to recover and left for the appropriate amount of time before euthanasia.

#### In Vivo Luciferase Assay to Assess Efficacy of Compounds.

All mice were blinded, separated into treatment groups, and assigned a random number; images were taken, not knowing which number belonged to which experimental group. 5 days post-hydrodynamic delivery, mice had 150 ml of a 15-mg/ml d-luciferin solution delivered intraperitoneally. After a 5-minute incubation period, mice were placed inside the chamber of an IVIS-100 luciferase imaging camera (Caliper Life Sciences, Waltham, MA) and imaged with a 2-minute exposure time. Mice were dosed 8, 24, and 32 hours after acquiring the baseline image with either compound OX03050 (40 mg/kg), vehicle alone, or pravastatin (600 mg/kg), and an uninjected group was also included as a control. Forty-eight hours onward from the first image, the mice were imaged under the exact same conditions as before. Images were analyzed using LivingImage software (Caliper Life Sciences).

## Results

### 

#### Identification of Novel Molecules that Drive Expression of the Human LDLR Promoter via a Mechanism Distinct from Statins.

**Fig. 1.**
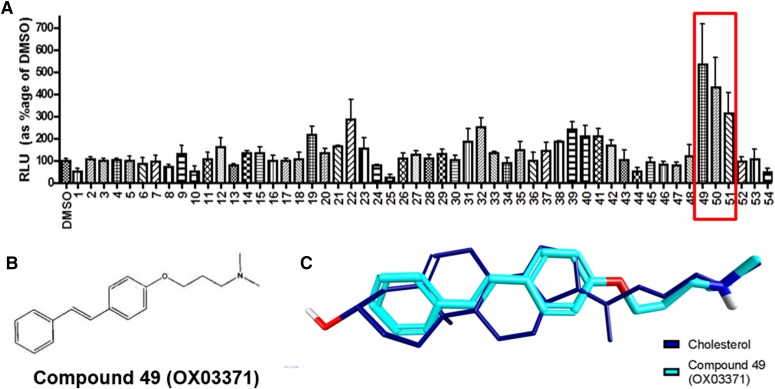
Identification of novel small molecules that upregulate *LDLR* genomic DNA promoter activity. A compound library of 216 small molecules was screened at a single concentration (20 *µ*M) in a CHO-p*LDLR-Luc* cell line. Luciferase is under the control of 10 kb genomic DNA upstream of the *LDLR* locus, including the promoter and elements essential for physiologic regulation. (A) Three initial hits appeared to give an increase in luciferase expression compared with DMSO-treated (0.1%) control cells. (B) The structure of compound 49 (OX03771), the most potent of the initial hits. (C) The structure of compound 49 (OX03771) and cholesterol, showing their similarity in structure. DMSO, dimethylsulfoxide.

**Fig. 2.**
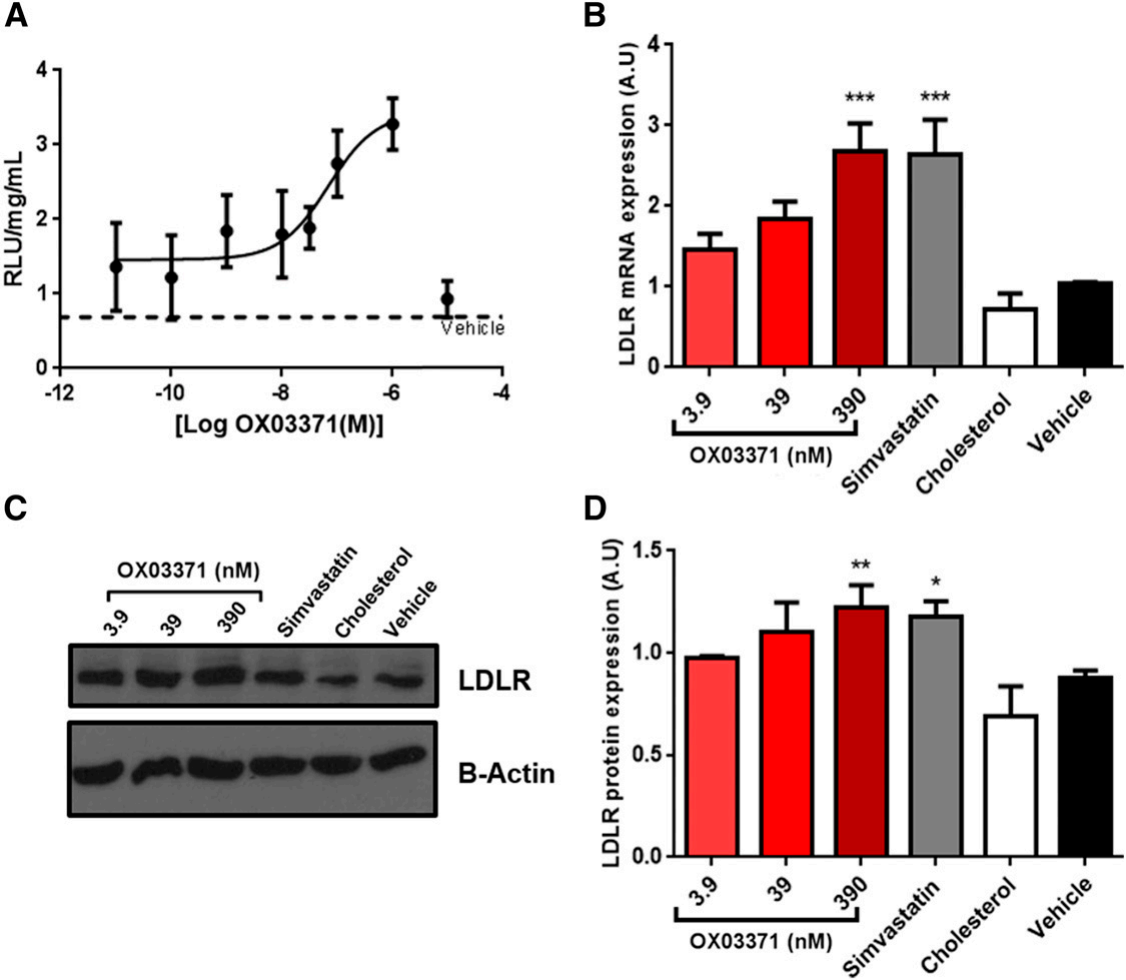
Compound OX03771 dose-dependently increases the LDLR at the mRNA and protein levels with an EC_50_ in the nanomolar range. (A) CHO-p*LDLR-Luc* cells were treated with compound OX03771 or with vehicle control (0.1% DMSO) for 48 hours before luciferase expression was measured. Compound OX03771 gave a dose-dependent increase in luciferase expression compared with vehicle-treated cells and had an EC_50_ in the nanomolar range. Luciferase expression was normalized to total protein (*n* = 4). (B) Hep3B cells were treated with increasing doses of compound OX03771 for 24 hours before mRNA expression was analyzed. Cholesterol-treated (25.8 *µ*M) and simvastatin-treated (500 nM) cells acted as the negative and positive controls, respectively. Compound OX03771 gave a significant increase in *LDLR* mRNA expression compared with vehicle-treated cells (*n* = 5). (C and D) Representative Western blot (C) and quantification (D) of mouse Hepa1-6 cells treated with increasing concentrations of compound OX03771 for 48 hours before Ldlr protein expression was analyzed. Cholesterol (25.8 *µ*M) and simvastatin (500 nM) were used as the negative and positive controls, respectively. Compound OX03771 gave a significant increase in Ldlr protein expression compared with vehicle cells (*n* = 3). Error bars denote the S.D. Significance represents treatment compared with vehicle-treated control. *P < 0.05; **P < 0.01; ***P < 0.001 (one-way analysis of variance with Dunnett post hoc analysis). A.U., arbitrary unit.

A CHO clonal cell line was developed (CHO-p*LDLR-Luc*), which contains a 10-kb genomic DNA human *LDLR* promoter element driving luciferase. The p*LDLR-Luc* construct has previously been shown to contain the necessary elements for physiologic regulation of expression of the *LDLR* locus (Hibbitt et al., 2010). To identify compounds that could upregulate expression of the *LDLR*, we focused our screening efforts by selecting compounds in silico with scaffolds reminiscent of known gene transcription modulators (e.g., sterols and steroid-like structures). We refined our in-house compound collection to 216 small molecules that we screened at a single concentration (20 *µ*M) in the CHO-p*LDLR-Luc* cell line. Compounds 49 [OX03771; (*E*)-*N*,*N*-dimethyl-3-(4-styrylphenoxy)propan-1-amine], 50, and 51 gave the greatest increase in luciferase activity compared with the vehicle-treated control (Fig. 1A). This series of compounds was of interest due to their similarity in structure to sterols (Fig. 1B), as shown in the overlay of compound OX03771 with cholesterol (Fig. 1C). Authentic samples of the three initial hits were synthesized and tested for a dose-dependent response: compound OX03771 was determined to be the most potent (EC_50_ ± S.E.M. = 39 ± 29 nM; Fig. 2A). Hep3B cells were transfected with a p*CMV-Luc* plasmid and treated with compound OX03771 (Supplemental Fig. 1A). No significant difference was seen between vehicle-treated or compound OX03771-treated cells expressing p*CMV-Luc*, ruling out compound OX03771 causing an increase in luciferase activity through stabilizing or interacting with d-luciferin or luciferase, a commonly observed artifact in these reporter assays (Thorne et al., 2012). To confirm that compound OX03771 itself was not contributing to the luminescent readout, untransfected Hep3B cells were treated with vehicle or compounds, with no increase in luminescence detected above untreated cells (Supplemental Fig. 1B). An adenylate kinase activity assay carried out on cell culture medium, a measure of cytotoxicity, demonstrated a favorable safety profile of compound OX03771, with only the 10-*µ*M dose showing an increase compared with vehicle-treated control cells (Supplemental Fig. 1C). The cytotoxicity observed at 10 *µ*M with compound OX03771 correlates with the drop off in luciferase activity observed in the dose-response curve at the same concentration.

#### Compound OX03771 Upregulates the LDLR at the mRNA and Protein Levels.

Human and mouse hepatocyte cell lines were treated with compound OX03771 to determine whether it could upregulate LDLR mRNA and protein. The EC_50_ dose established in the CHO-p*LDLR-Luc* assay (39 nM) was chosen, along with doses 10-fold above and below (Fig. 2A). Hep3B cells were treated with compound OX03771, cholesterol (negative control), or a statin (positive control). Compound OX03771 gave a dose-dependent increase at the mRNA level with a significant increase at 390 nM (Fig. 2B). To confirm the increase in *LDLR* mRNA resulted in upregulation of the LDLR protein, Hepa1-6 cells were treated with the same concentrations of compound OX03771 (Fig. 2C). Compound OX03771 caused a significant upregulation of the LDLR protein compared with vehicle-treated control cells at 390 nM similar to that of simvastatin (Fig. 2D).

#### Compound OX03050 Is a More Potent Analog of Compound OX03771 with Improved Pharmacokinetic Properties.

**Fig. 3.**
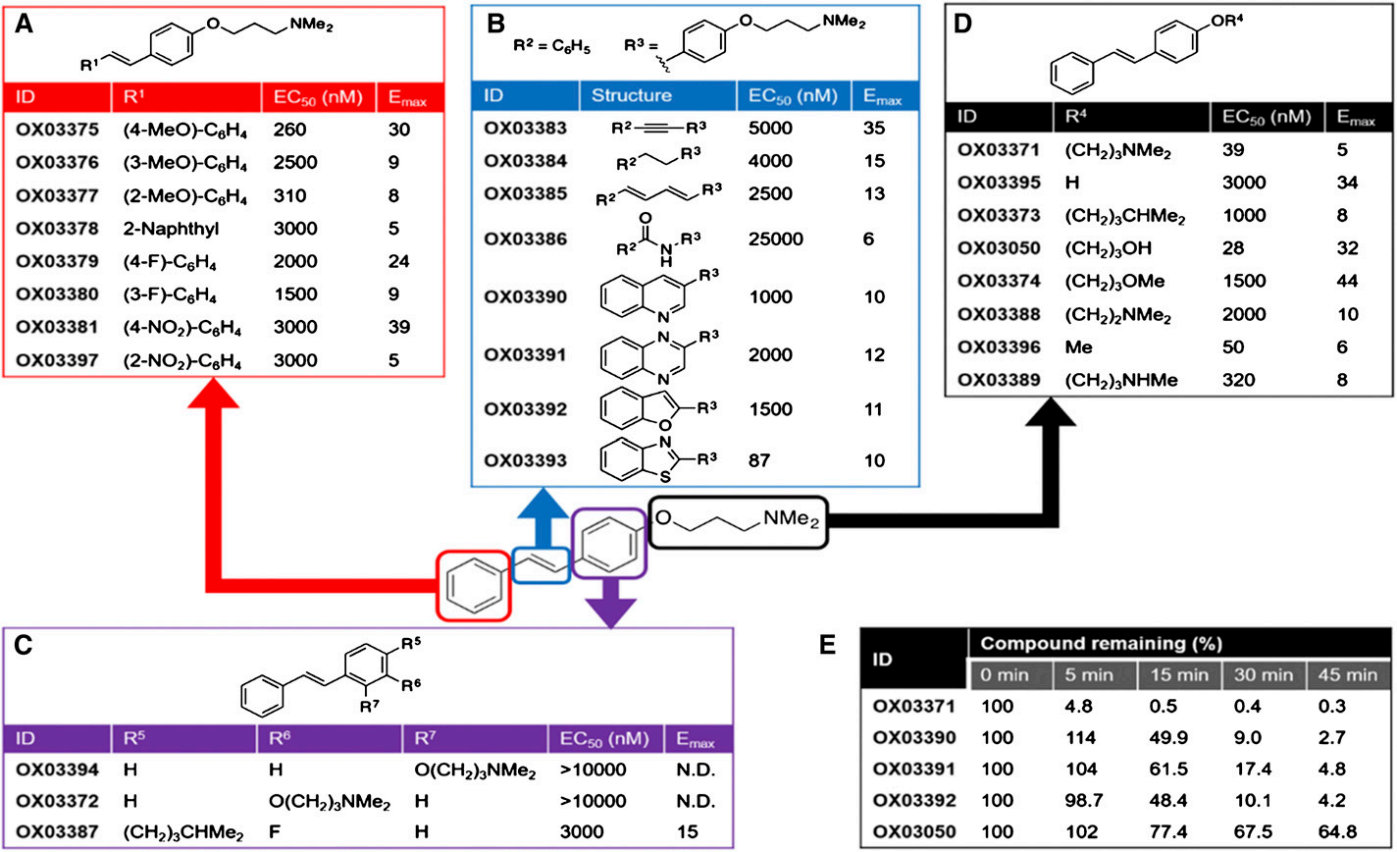
Identification of compound OX03050, a more potent analog of compound OX03771 with improved metabolic stability. Systematic SAR studies were carried out on compound OX03771, whereby each component of the molecule was altered individually and screened in CHO-p*LDLR-Luc* cells at concentrations of 1, 10, 30, 100, 300, 1000, and 10,000 nM. (A–D) The red squared aromatic ring (A), blue squared double bond linker (B), purple squared aromatic ring (C), and *N,N*-dimethylaminopropyloxy chain modifications (D) led to the identification of compound OX03050, a compound with improved potency. All compounds were screened in duplicate. (E) Listed compounds were spiked into mouse liver microsomes; after the time points given, the remaining amounts were quantified through liquid chromatography/mass spectrometry. The amount is given in the table as a percentage in comparison with the amount detected at the 0-minute time point. Of these, compound OX03050 was the most stable, with over half still present after 45 minutes.

Compound OX03771 was found to have a half-life of less than 5 minutes when incubated with mouse liver microsomes and quantified using liquid chromatography/mass spectrometry (Supplemental Fig. 2A). To improve the efficacy, pharmacokinetic profile, and in vitro absorption, distribution, metabolism, and excretion (ADME) of compound OX03771, analogs were made and their efficacy was tested in the CHO-p*LDLR-Luc* clonal cell line (Fig. 3). Microsomal incubation of selected analogs was also undertaken to assess metabolic stability. Compound OX03371 was conceptually split into four key regions: the two aromatic rings in the stilbene (red and purple rectangles in Fig. 3, A and C, respectively), the carbon-carbon double bond linker (blue rectangle in Fig. 3B), and the *N*,*N*-dimethylaminopropyloxy chain (black rectangle in Fig. 3D). Each of the four regions was modified systematically to assess SARs. The compounds developed in each series were tested to obtain EC_50_ and *E*_max_ values. All modifications to aromatic ring A tested (Fig. 3A), including the introduction of electron- withdrawing or electron-donating substituents, led to a reduction in activity compared with compound OX03771 (EC_50_ = 39 nM, *E*_max_ = 5).

To alter the three-dimensional shape of the molecule, the alkane, alkyne, and (*E*,*E*)-1,3-butadiene linker analogs were synthesized (Fig. 3B), thereby varying either the compound’s conformational flexibility or the relative orientation of or distance between the two aryl units. A secondary amide analog was also prepared as an isostere of the (*E*)-alkene. Compound OX03384 [*N*,*N*-dimethyl-3-(4-phenethylphenoxy)propan-1-amine], which contains a fully saturated alkyl linker group, alkyne OX03383 [*N*,*N*-dimethyl-3-(4-(phenylethynyl)phenoxy)propan-1-amine], and butadiene OX03385 [*N*,*N*-dimethyl-3-(4-((1*E*,3*E*)-4-phenylbuta-1,3-dien-1-yl)phenoxy)propan-1-amine] all resulted in lower potency, suggesting that the geometrical constraints imparted by the (*E*)-olefin are important. Compound OX03386 [*N*-(4-(3-(dimethylamino)propoxy)phenyl)benzamide], containing a secondary amide linker, also showed much lower potency. Given the previously reported propensity of olefins to undergo metabolic oxidation reactions (Dharwadkar, 2016), it was also of interest to investigate bicyclic isosteres OX03390-93, some of which may be anticipated to be more metabolically stable. Quinoline OX03390 [*N*,*N*-dimethyl-3-(4-(quinolin-3-yl)phenoxy)propan-1-amine], quinoxaline OX03391 [*N*,*N*-dimethyl-3-(4-(quinoxalin-2-yl)phenoxy)propan-1-amine], and benzofuran OX03392 [3-(4-(benzofuran-2-yl)phenoxy)-*N*,*N*-dimethylpropan-1-amine] all showed a reduction in potency compared with compound OX03771. Encouragingly, however, benzothiazole OX03393 [3-(4-(benzo[*d*]thiazol-2-yl)phenoxy)-*N*,*N*-dimethylpropan-1-amine] showed only a modest drop in potency, compared with compound OX03771, and a similar *E*_max_.

Altering the regiochemistry within the aromatic ring C was found to have a profound effect on the potency: both *ortho* and *meta*-*N*,*N*-dimethylaminopropyloxy substituted analogs, compounds OX03394 [(*E*)-*N*,*N*-dimethyl-3-(2-styrylphenoxy)propan-1-amine] and OX03373 [(*E*)-1-((4-methylpentyl)oxy)-4-styrylbenzene], showed a complete loss of activity (Fig. 3C). Compound OX03387 [(*E*)-3-(2-fluoro-4-styrylphenoxy)-*N*,*N*-dimethylpropan-1-amine] suggested that reducing the electron density of the aromatic ring C through the introduction of an additional fluoro substituent resulted in reduced activity.

Finally, the *N*,*N*-dimethylaminopropyloxy chain was modified as shown in Fig. 5D. The hydroxyaryl derivative, compound OX03395 [(*E*)-4-styrylphenol], lacking the *N*,*N*-dimethylaminopropyl side chain showed a reduction in potency (EC_50_ = 3000 nM). The monomethyl substituted counterpart (compound OX03389 [(*E*)-*N*-methyl-3-(4-styrylphenoxy)propan-1-amine]) gave an apparent modest drop in potency (EC_50_ = 320 nM) compared with compound OX03771, whereas the truncated homolog (compound OX03388 [(*E*)-*N*,*N*-dimethyl-2-(4-styrylphenoxy)ethanamine) showed a significant reduction in potency (EC_50_ = 2000 nM). Compound OX03050, containing a propyl alcohol chain, gave a comparable EC_50_ of 26 nM compared with 39 nM (compound OX03771) and a 6-fold higher *E*_max_, whereas both the *O*-methyl ether analog (compound OX03374 [(*E*)-1-(3-methoxypropoxy)-4-styrylbenzene], EC_50_ = 1500 nM) and the alkyl substituted analog (compound OX03373, EC_50_ = 1000 nM) showed a reduction in activity. Given that, under the assay conditions, the amines would exist predominantly as their corresponding ammonium salts; taken together, these data suggest that the presence of a hydrogen bond donor within the side chain may confer higher levels of potency.

Analogs of OX03371, that were predicted to improve the ADME profile of the compound, including OX03050, were incubated with mouse liver microsomes to measure their metabolic conversion over time. (Fig. 3E). Of these, compound OX03050 demonstrated the greatest metabolic stability, having a half-life greater than 45 minutes. All of the other compounds tested had dropped below 5% of their starting amount by 45 minutes; therefore, with the greatest potency and best in vitro metabolic stability profile, compound OX03050 was taken forward for in vivo testing.

#### Compound OX03050 Is Able to Drive the Human *LDLR* Promoter In Vivo.

**Fig. 4.**
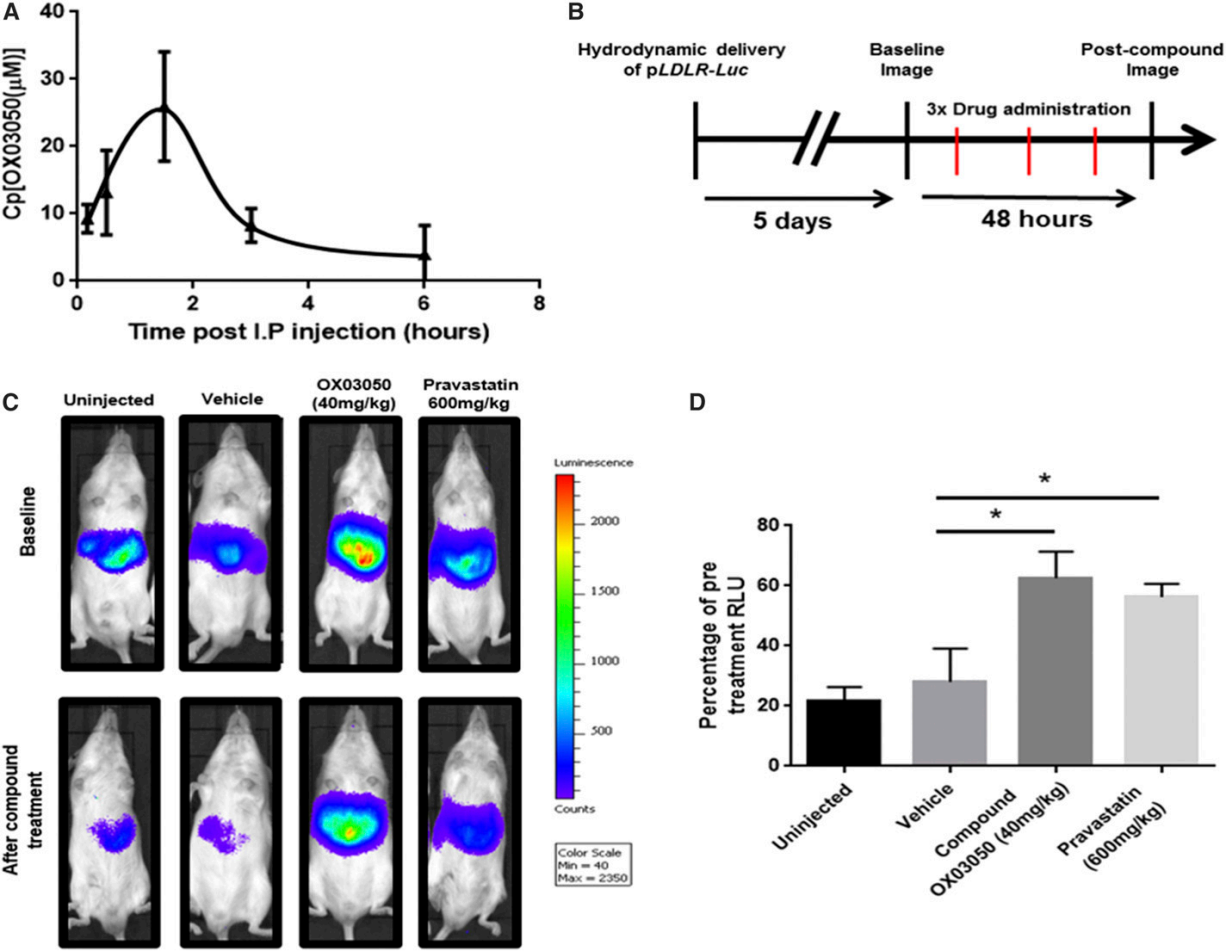
Compound OX03050 is able to increase expression of the human *LDLR* promoter in a mammalian system. (A) Compound OX03050 (40 mg/kg) was detectable up to 6 hours after intraperitoneal administration in mouse plasma. (B) Timeline of the in vivo efficacy study: p*LDLR-Luc* plasmid was delivered to MF-1 male mice via hydrodynamic injection on day 0. (C) Five days after hydrodynamic injection, a baseline image was taken for each mouse. Three doses of either vehicle, compound OX03050 (40 mg/kg), or pravastatin (600 mg/kg) were administered over 48 hours before a second image was taken. An uninjected group, where the plasmid was delivered but no compound was administered, was used as a negative control. (D) Compound OX03050 and pravastatin gave a significant increase in luciferase expression compared with vehicle-treated mice. *P < 0.05 (*n* = 5 to 6 per group; one-way analysis of variance with Dunnett post hoc analysis). Error bars denote S.E.M.

To test whether the ADME profile seen in mouse liver microsomes was confirmed in vivo, an HPLC assay was developed to measure compound OX03050 in mouse plasma. Forty milligrams per kilogram of compound OX03050 was delivered intraperitoneally to CD-1 male mice and plasma was taken at various time points up to 6 hours (Fig. 4A). Compound OX03050 demonstrated a half-life of 84 minutes in vivo, consistent with the in vitro stability data, and could be detected at least up to 6 hours after dosing. To test the pharmacodynamic effects of compound OX03050 in vivo, MF-1 male mice received a hydrodynamic injection of the p*LDLR-Luc* plasmid to quantify *LDLR* promoter activity in vivo by noninvasive bioluminescence imaging. Baseline images were obtained 5 days after injection of the p*LDLR-Luc* plasmid (Fig. 4C). Mice then received three doses of either vehicle, compound OX03050 (40 mg/kg), or pravastatin (600 mg/kg) spaced over 48 hours before final luciferase imaging (Fig. 4C). An uninjected group that received the p*LDLR-Luc* plasmid but no intraperitoneal injections was used as a negative control. Compound OX03050–injected mice had significantly higher luciferase expression after compound delivery compared with vehicle-treated mice (Fig. 4B). Pravastatin-injected mice also had significantly higher luciferase expression than vehicle-injected mice, as would be expected and has previously been shown (Hibbitt et al., 2010). No difference was observed between uninjected mice and mice receiving the vehicle treatment. Here we demonstrate that compound OX03050 is able to drive expression of the human *LDLR* promoter in vivo.

#### Compounds OX03050 and OX03771 Inhibit Squalene Synthase.

**Fig. 5.**
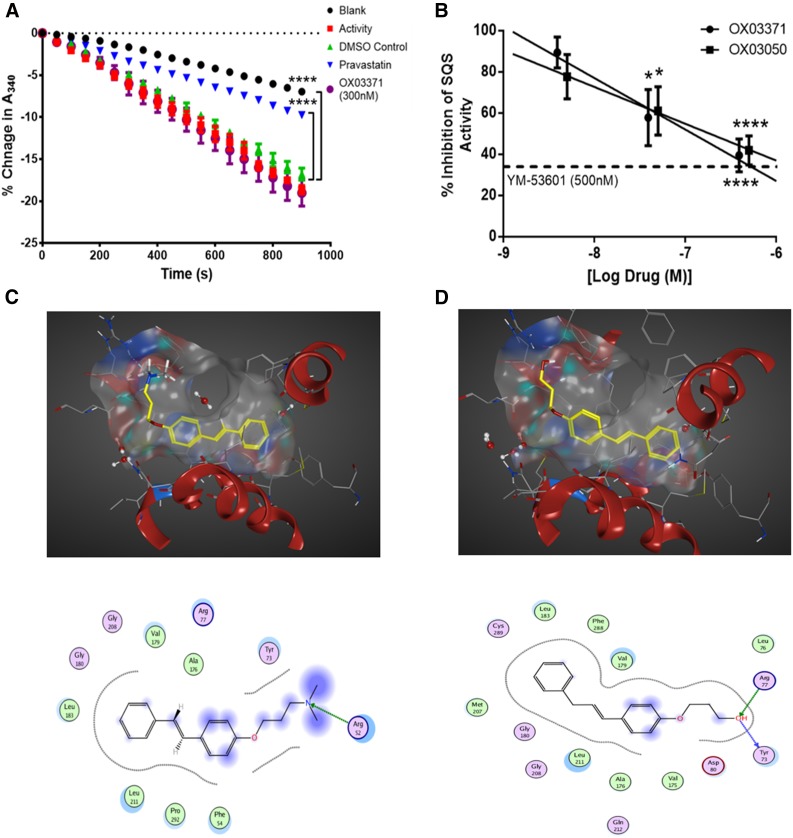
Compounds OX03771 and OX03050 competitively inhibit squalene synthase through binding in the active site. (A) Purified HMGCR was added to samples containing either vehicle, pravastatin, or compound OX03771, alongside all substrates necessary for the reaction to process. No enzyme added (blank) and no compound added (activity) samples acted as negative controls. Pravastatin significantly inhibited the decrease in absorbance at 340 nm, representing NADPH being oxidized to NADP^+^ and indicative of HMGCR activity. None of the other three compounds inhibited this decrease, demonstrating that they do not inhibit HMGCR (*n* = 3). Error bars denote the S.D. (B) Purified human liver microsomes were incubated with radiolabeled FPP and all necessary cosubstrates in the presence of compounds OX03771 or OX03050 or vehicle. Both compounds OX03771 and OX03050 dose-dependently inhibited squalene synthase activity at nanomolar concentrations (*n* = 4–7). Error bars denote the S.E.M. (C and D) In silico modeling of compounds OX03771 (C) and OX03050 (D) using MOE software. *P < 0.05; ****P < 0.0001.

We next confirmed that compound OX03771 upregulates the LDLR through a mechanism distinct from statins (Fig. 5A). The activity of purified HMGCR is measured indirectly through measuring the absorption of NADPH at 340 nm over time. As HMGCR converts HMG-CoA to mevalonate, NADPH (a cosubstrate in the reaction) is oxidized to NADP^+^, leading to a decrease in the absorption at 340 nm. The activity of the enzyme was confirmed, as there was a significant decrease in absorption between blank (no enzyme added) and vehicle-treated samples (enzyme added). Pravastatin was used a positive control, which significantly inhibited the decrease in absorption compared with a vehicle-treated sample after 15 minutes. No significant difference was observed between compound OX03771- and vehicle-treated samples (Fig. 5A), demonstrating that compound OX03771 does not exert its effects on LDLR expression via inhibition of HMGCR.

Squalene synthase activity was then measured in human liver microsomes in the presence of compounds OX03050 and OX03771. The compounds were preincubated with the microsomes for 10 minutes before the reaction was started by adding [^3^H]-FPP. The reaction went on for 10 minutes before inhibition was assessed. The EC_50_ values for compound OX03050 (26 nM) and compound OX03771 (39 nM) established in the CHO-p*LDLR-Luc* assay and concentrations 10-fold above and below this were used. Compounds OX03050 and OX03771 both inhibited the conversion of FPP to squalene in a dose-dependent manner, with both showing approximately 60% inhibition at the higher dose (Fig. 5B). This suggests that the increase in LDLR expression observed with both compounds OX03050 and OX03771 is mediated, at least in part, by inhibition of squalene synthase.

In silico modeling was carried out on these compounds in an effort to rationalize their inhibitory potencies against squalene synthase. MOE software Chemical Computing Group Inc. Montreal, Quebec, Canada was used with the available human squalene synthase crystal structure (Protein Data Bank identifier 1EZF), which had been cocrystallized with the ligand CP-320473 [(2*S*)-2-[[2-[(3*R*,5*R*)-7-chloro-1-(2,2-dimethylpropyl)-5-naphthalen-1-yl-2-oxo-5*H*-4,1-benzoxazepin-3-yl]acetyl]amino]butanedioic acid] (Pandit et al., 2000). Compound OX03771 was docked in both its neutral state and as the corresponding protonated ammonium species for comparison. It was anticipated that OX03371 would exist predominantly as the protonated form under physiologic conditions, but it was unclear which form would preferentially bind to squalene synthase. Docking studies were conducted as 30 independent experiments. No consistent docking prediction was found for the ammonium salt of OX03771. In contrast, all solutions for the free base of OX03771 were predicted to bind in a similar manner to the ligand CP-320473 in the active site, with the hydrophobic aryl group sitting deep in the pocket while the chain bearing the hydrogen bond acceptor amino group was predicted to protrude out to interact with Arg77 (Fig. 5C). To further explore this putative interaction, compound OX03394 was docked into the active site, a molecule that demonstrated no activity in the CHO-p*LDLR-Luc* assay (Supplemental Fig. 2). As with OX03771, the amino group within OX03994 is predicted to interact with Arg77 (Supplemental Figure 3); however, the molecule is predicted to adopt an alternative orientation within the active site as the regiochemistry of the *N*,*N*-dimethylaminopropyloxy substituent is switched from the *para* to *meta*. It is possible that this leads to greater instability of the complex, which could explain the lack of activity observed for this compound. Compound OX03050 was predicted to bind in a similar manner to compound OX03771 (Fig. 4D). As with the previous compounds, the hydrophobic aryl group was predicted to insert into the active site pocket but in a slightly different conformation, with the terminal hydroxyl group predicted to interact with Arg77 and Tyr72. The squalene synthase activity assay coupled with the in silico modeling provides a potential rationale to support compound OX03050 and OX03771 as inhibitors of squalene synthase.

#### A Squalene Synthase Inhibitor and a Statin in Combination Cause a Synergistic Drive in LDLR Expression.

**Fig. 6.**
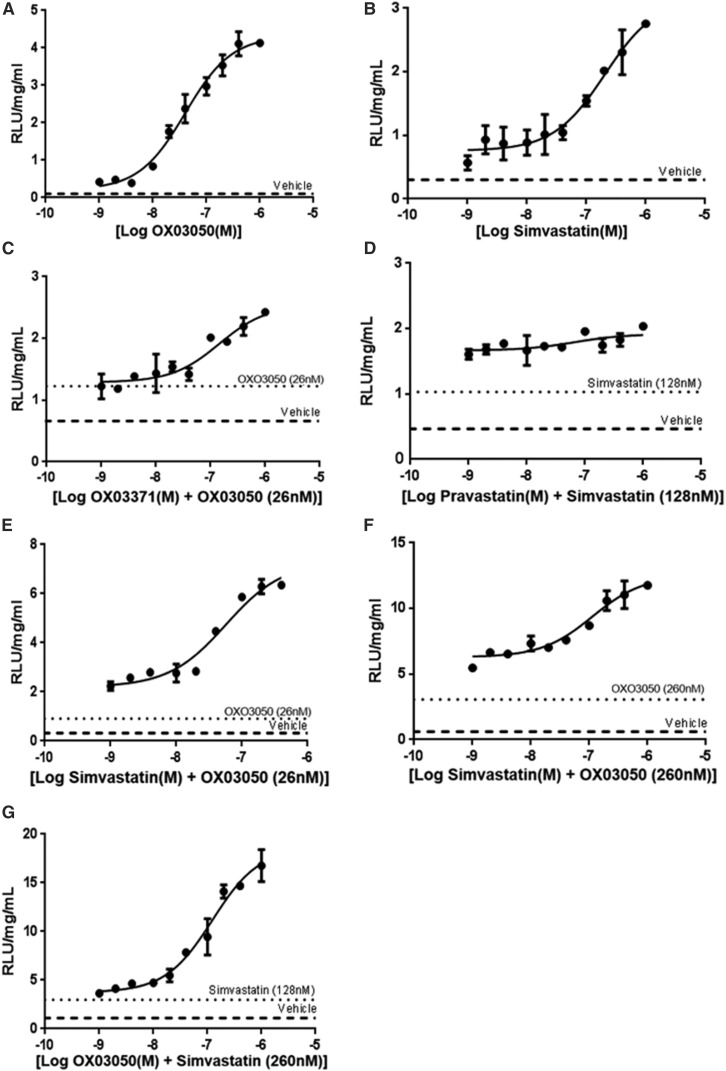
A statin and a squalene synthase inhibitor produce a synergistic effect, further upregulating *LDLR* promoter activity. To determine whether a statin and a squalene synthase inhibitor produced a greater upregulation of the *LDLR* promoter, dose-response experiments in the CHO-p*LDLR-Luc* cell line were carried out in the presence of the different cholesterol biosynthesis inhibitors. (A and B) A dose response of compound OX03050 (A) and simvastatin (B) established the EC_50_ and EC_80_ values to be used in the combination treatments. (C) A dose-response of compound OX03771 in the presence of the EC_50_ of compound OX03050, looking at the effect of two squalene synthase inhibitors. (D) A dose response of pravastatin in the presence of the EC_50_ of simvastatin, looking at the effect of two statins. (E and F) The EC_50_ (E) and EC_80_(F) of compound OX03050 treated alongside a dose response of simvastatin. (G) The EC_50_ of simvastatin treated alongside a dose response of compound OX03050. The dashed line represents vehicle-treated (0.1% DMSO) cells and the dotted line is the single concentration of the EC_50_ or EC_80_ of the compound stated on the figure. In combination using a statin and squalene synthase inhibitor leads to a much larger increase in luciferase expression than can be seen with two classes of the same inhibitor (*n* = 4). Error bars denote the S.D.

We sought to determine whether a squalene synthase inhibitor and statin used in combination are able to drive higher levels of human *LDLR* promoter expression in a synergistic manner. The CHO-p*LDLR-Luc* cell line was treated with compounds for 48 hours to evaluate the effect of these small molecules in combination to drive the human *LDLR* promoter. A dose-response curve was generated for compound OX03050 (Fig. 6A) and simvastatin (Fig. 6B) alone, giving EC_50_ values of 26 and 128 nM, respectively. A dose-response of compound OX03771 was carried out in the presence of compound OX03050 dosed at its EC_50_ (Fig. 6C). There was no enhancement in luciferase activity using two squalene synthase inhibitors (*E*_max_ = 2.43 RLU/mg per ml) compared with the dose response of compound OX03771 alone (*E*_max_ = 3.28 RLU/mg per ml) (Fig. 2A). The same effect was seen when two statins were dosed together; a dose response of pravastatin was carried out in the presence of simvastatin dosed at its EC_50_ (Fig. 6D). No increases in the luciferase activity were seen using two statins (*E*_max_ = 2.04 RLU/mg per ml) compared with simvastatin alone (*E*_max_ = 2.76 RLU/mg per ml).

However, dose-response curves for simvastatin in the presence of compound OX03050 dosed at its EC_50_ (Fig. 6E) and EC_80_ (Fig. 6F) gave a much greater increase in luciferase activity, giving *E*_max_ values of 6.35 and 11.77 RLU/mg per ml, respectively, compared with two statins alone. The same was also true for the reverse experiment in which a dose-response of compound OX03050 was carried out in the presence of simvastatin dosed at its EC_50_ (Fig. 6G). The *E*_max_ value was 16.77 RLU/mg per ml, a much larger increase in luciferase activity than seen using two squalene synthase inhibitors in combination. The fold increases in activity given when two squalene synthase inhibitors or two statins were dosed compared with vehicle-treated cells were 3.9 and 1.1, respectively. In comparison, the fold increases when a statin was dosed alongside a squalene synthase inhibitor were 10- and 16.1-fold compared with vehicle-treated cells. This is consistent with a synergistic effect of inhibiting the cholesterol biosynthesis pathway at two distinct parts of the pathway, which can greatly enhance the expression of the human LDLR.

## Discussion

Here we present a novel series of small molecules able to upregulate LDLR expression through inhibition of squalene synthase. We first identified compound OX03771 from a focused small compound screen (216 molecules) in a reporter cell line produced in our laboratory. The reporter line contains a 10-kb human *LDLR* promoter element, including all components required for physiologic regulation of expression, driving luciferase. The initial hit OX03771 was then confirmed in two secondary assays and was shown to increase LDLR expression at both the mRNA and protein levels. Through systematic SAR studies, we identified an even more potent analog of compound OX03771 with improved in vitro metabolic stability (compound OX03050). We dosed compound OX03050 in the mouse, establishing a pharmacokinetic profile of the drug, and demonstrated that the efficacy seen in vitro translates to an in vivo system. Furthermore, we used a unique approach to quickly assess whether compound OX03050 can drive the human LDLR promoter in a mammalian system. Hydrodynamic delivery (a well established method to deliver plasmid DNA directly to the liver; Liu et al., 1999) of the reporter vector resulted in an efficient and quick way to assess in vivo efficacy of our small molecules.

We next elucidated the mechanism of action of compounds OX03771 and OX03050 as inhibiting the enzyme squalene synthase, the first committed step in cholesterol biosynthesis. This suggests that the increase in LDLR expression observed with both compounds OX03050 and OX03771 is mediated, at least in part, by inhibition of squalene synthase. Other inhibitors of squalene synthase have previously been reported and investigated for their effects on lipid lowering (Elsayed and Evans, 2008). Some of our small molecules resemble a series of stilbene derivatives reported several years ago as inhibitors of squalene synthase with modest potencies (Harwood et al., 1997). All of the examples described by Harwood et al. (1997) incorporate a basic amino group within the side chain appended to the stilbene, and they propose that the corresponding ammonium derivatives act as structural mimics of the cationic intermediate produced within the enzyme active site upon farnesyl pyrophosphate dimerization. Intriguingly, our compounds retain inhibitory potency even in the absence of an amino group in the side chain; for example, OX03050 shows similar potency to OX03371, and our modeling studies predict similar interactions between protein and inhibitor for both compounds. Our small molecules share some similarity in structure to cholesterol, so it is possible that they may also interact with other enzymes downstream of squalene synthase; however, this must be further investigated. Finally, we provided data to support that a squalene synthase inhibitor used in conjunction with a statin acts synergistically to increase LDLR expression.

In the future, our in vivo reporter model could be used to screen further arrays of small molecules for their ability to drive the human *LDLR* promoter in a mammalian system. This platform provides a quick and simple method to eliminate any compounds that fail to carry in vitro efficacy to an in vivo system. It is well known that statins do not have the same effect in rodents as they show in humans, with studies showing a decrease in LDLR expression and no change in total cholesterol (Rashid et al., 2005) after statin administration. This could be attributed in part to the difference in how rodents and humans carry their cholesterol (Camus et al., 1983), with mice carrying most of their cholesterol in the high-density lipoprotein fraction. Other differences, due to the absence of certain proteins such as cholesteryl ester transfer protein (Guyard-Dangremont et al., 1998) in mouse or inclusion of apolipoprotein B-48 (Greeve et al., 1993) into LDL particles result in a decreased shuttling of cholesterol ester to the LDL particles and enhanced clearance of LDL particles, respectively. Inhibitors of cholesterol biosynthesis only consistently show efficacy in larger mammals such as the guinea pig (Matsunaga et al., 1991) or rabbit (Krause and Newton, 1995). Our reporter system could provide a more economical and higher-throughput platform to assess in vivo efficacy before embarking on a longer-term efficacy study in a larger animal model.

As lipid goals become stricter for individuals with dyslipidemia, especially high-risk patients such as those with heterozygous familial hypercholesterolemia (heFH), high-dose statins may become intolerable. Many studies have shown that only a small percentage of patients with heFH reach their lipid goal (Pijlman et al., 2010). The emergence of proprotein convertase subtilisin/kexin type 9 inhibitors is a welcome addition to try and further lower the LDL cholesterol of patients with heFH (Robinson et al., 2015). The excellent lipid lowering seen with this class of biologics is surely promising; however, because these drugs must be administered via a subcutaneous injection every 2 weeks and are expected to have a high cost (Zimmerman, 2015), an oral medication that can be taken alongside lower-dose statins may still be of great use. We demonstrated that a squalene synthase inhibitor with a statin could cause a greater upregulation of *LDLR* promoter activity than can be achieved with either class of drug alone. Therefore, a lower dose of both classes of drugs could be used in high-risk patients who are unable to tolerate maximal dose statins, with the hope of alleviating the side effects these patients experience. This enhanced efficacy has been shown in the clinic previously (Stein et al., 2011); however, the liver side effects seen with the squalene synthase inhibitor may mean that a statin will have to be used alongside any squalene synthase inhibitor to inhibit a build up of FPP (Wasko et al., 2011). Squalene synthase inhibitors have also been shown to lower triglycerides (Ugawa et al., 2000), which may be explained by recent studies investigating the orthologs differentially affected by squalene synthase inhibitors and statins (Rondini et al., 2016). Only squalene synthase inhibitors were shown to alter cellular lipid metabolism and thus may provide another added benefit to using them alongside a statin.

Here, we have characterized a novel series of small molecules, the most active of which have potency in the nanomolar range. To further evaluate the LDL-cholesterol lowering potential of these compounds, the next step would be to dose in a larger animal model, such as the guinea pig or rabbit model. These larger species share a similar lipid profile to humans, in which the majority of cholesterol is carried in the LDL fraction and cholesterol levels respond to compounds that inhibit the cholesterol biosynthesis pathway. This further assessment will be critical for the progression of this series of molecules toward any future therapy.

## Abbreviations

ADME: absorption, distribution, metabolism, and excretion
CHO: Chinese hamster ovary
CP-320473: (2*S*)-2-[[2-[(3*R*,5*R*)-7-chloro-1-(2,2-dimethylpropyl)-5-naphthalen-1-yl-2-oxo-5*H*-4,1-benzoxazepin-3-yl]acetyl]amino]butanedioic acid
FPP: farnesyl diphosphate
heFH: heterozygous familial hypercholesterolemia
HMGCR: 3-hydroxy-3-methylglutaryl coenzyme A reductase
HPLC: high-performance liquid chromatography
LDL: low-density lipoprotein
LDLR: low-density lipoprotein receptor
OX03050: (*E*)-3-(4-styrylphenoxy)propan-1-ol
OX03373: (*E*)-1-((4-methylpentyl)oxy)-4-styrylbenzene
OX03374: (*E*)-1-(3-methoxypropoxy)-4-styrylbenzene
OX03383: *N*,*N*-dimethyl-3-(4-(phenylethynyl)phenoxy)propan-1-amine
OX03384: [*N*,*N*-dimethyl-3-(4-phenethylphenoxy)propan-1-amine
OX03385: *N*,*N*-dimethyl-3-(4-((1*E*,3*E*)-4-phenylbuta-1,3-dien-1-yl)phenoxy)propan-1-amine
OX03386: *N*-(4-(3-(dimethylamino)propoxy)phenyl)benzamide
OX03387: (*E*)-3-(2-fluoro-4-styrylphenoxy)-*N*,*N*-dimethylpropan-1-amine
OX03388: (*E*)-*N*,*N*-dimethyl-2-(4-styrylphenoxy)ethanamine
OX03389: (*E*)-*N*-methyl-3-(4-styrylphenoxy)propan-1-amine
OX03390: *N*,*N*-dimethyl-3-(4-(quinolin-3-yl)phenoxy)propan-1-amine
OX03391: *N*,*N*-dimethyl-3-(4-(quinoxalin-2-yl)phenoxy)propan-1-amine
OX03392: 3-(4-(benzofuran-2-yl)phenoxy)-*N*,*N*-dimethylpropan-1-amine
OX03393: 3-(4-(benzo[*d*]thiazol-2-yl)phenoxy)-*N*,*N*-dimethylpropan-1-amine
OX03394: (*E*)-*N*,*N*-dimethyl-3-(2-styrylphenoxy)propan-1-amine
OX03395: (*E*)-4-styrylphenol
OX03771: (*E*)-*N*,*N*-dimethyl-3-(4-styrylphenoxy)propan-1-amine
RLU, Relative light units: SAR, structure-activity relationship
TFA: trifluoroacetic acid
